# Traumatic pancreatic duct injury successfully treated with endoscopic retrograde cholangiopancreatography and stent placement in a 6-year-old boy

**DOI:** 10.1055/a-2376-5892

**Published:** 2024-08-13

**Authors:** Huma Asmat, Usama Al Farsi, Phillip Harrison, Deepak Joshi

**Affiliations:** 1 111990 Department of Hepatology and Hepatobiliary Medicine, Kingʼs College Hospital, London, United Kingdom of Great Britain and Northern Ireland

Pancreatic injury in children is rare, accounting for less than 1% of all pediatric traumas, but it does carry significant morbidity and mortality 1 .

Herein we report a case of a high grade pancreatic injury in a child successfully managed with a minimally invasive endoscopic approach alone without the need of surgical intervention.

A six-year-old boy presented to the hospital with abdominal pain and vomiting after sustaining a handlebar injury while riding a bike. On arrival, he was hemodynamically stable and afebrile. He had a bruise on the upper abdomen. There were no signs of peritonism. Biochemistry showed elevated levels of amylase and lipase at 454 U/L (normal range 28–100 U/L) and 514 U/L (normal range 13–60 U/L), respectively. Axial imaging ( Fig. 1 , Fig. 2 ) demonstrated a fracture of the main pancreatic duct in the body of the pancreas.

**Fig. 1:**
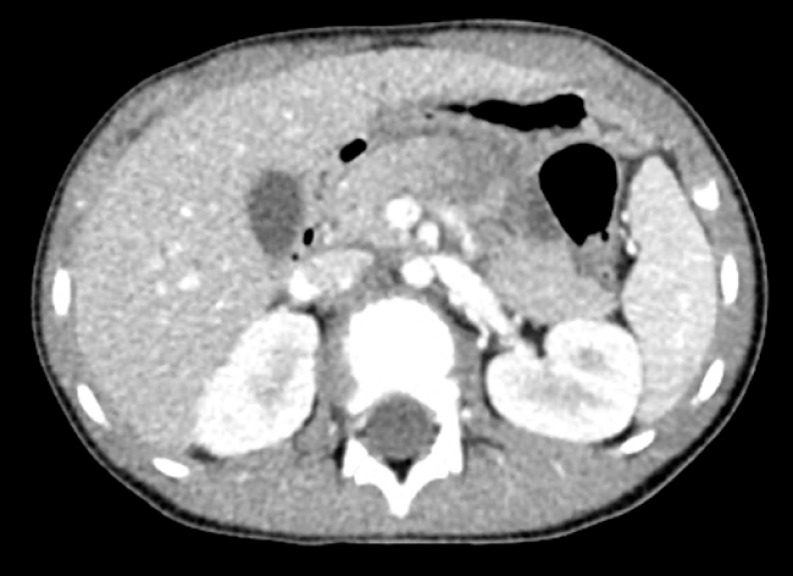
Computed tomography scan of abdomen (cross-sectional view) demonstrating pancreatic hematoma and edema suggestive of laceration of the pancreas.

**Fig. 2:**
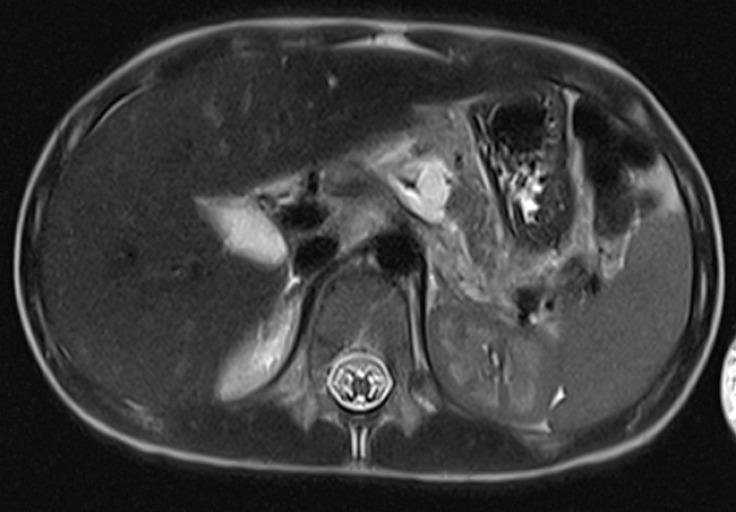
Magnetic resonance imaging (MRI) of the abdomen demonstrating pancreatic duct fracture draining into the fluid collection in the fracture plane in the body of the pancreas.

Endoscopic retrograde cholangiopancreatography (ERCP) was performed on day 4 of presentation under general anesthesia ( Video 1 ). The major papilla was cannulated with a GT-1-T cannula and Terumo 0.035 angled tip guidewire. The pancreatogram demonstrated a leak in the body of the pancreas ( Fig. 3 ), but the distal PD was delineated. The guidewire was manipulated and advanced across the fracture followed by the GT-1-T cannula. The Terumo guidewire was then exchanged for a 0.025 VisiGlide guidewire. A 7-cm, 5-Fr single-pigtail plastic stent ( Fig. 4 ) was placed successfully across the leak.

**Fig. 3:**
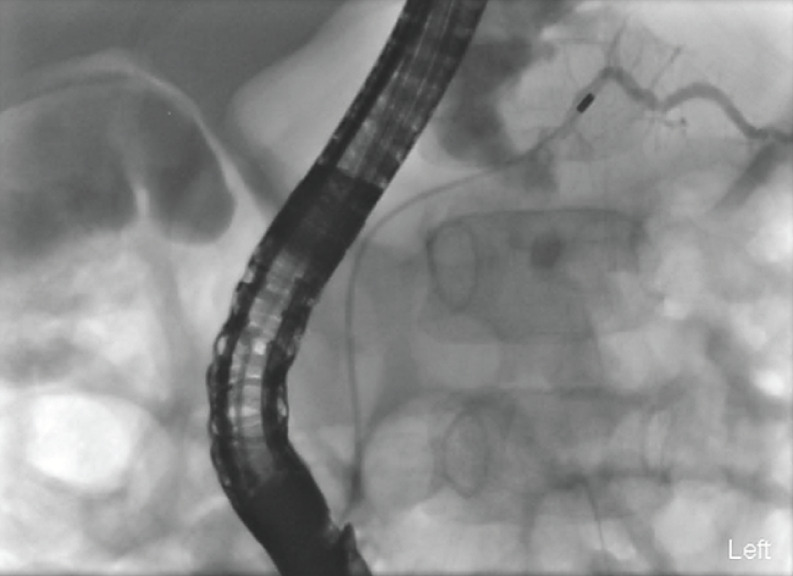
Fluoroscopy image during endoscopic retrograde cholangiopancreatography (ERCP) demonstrating pancreatogram with fracture and leak of the main pancreatic duct.

**Fig. 4:**
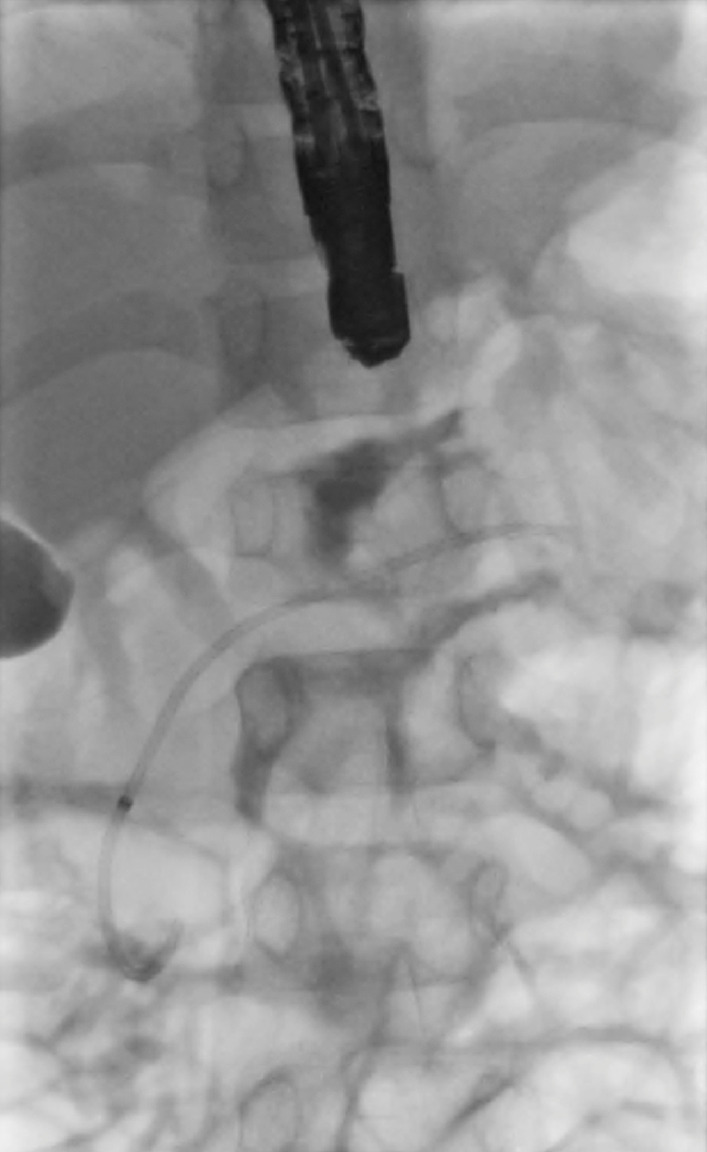
Fluoroscopy image during ERCP showing a 7-cm, 5-Fr single-pigtail plastic stent placed across the leak.

Fluoroscopy during endoscopic retrograde cholangiopancreatography demonstrating cannulation of main pancreatic duct, followed by contrast injection confirming pancreatic duct fracture and leak. A 7-cm, 5-Fr single-pigtail plastic stent was placed successfully across the fracture site.Video 1

The patient’s pain improved 48 hours post-ERCP and he was discharged home. An abdominal ultrasound ( Fig. 5 ) after 1 month did not show any significant intra-abdominal collection. A repeat ERCP was performed after 3 months and did not show a leak or stricture. A 6-Fr, 6-cm Archimedes fast degradation biodegradable stent was inserted prophylactically into the main PD. The child remains well 1 year later.

**Fig. 5:**
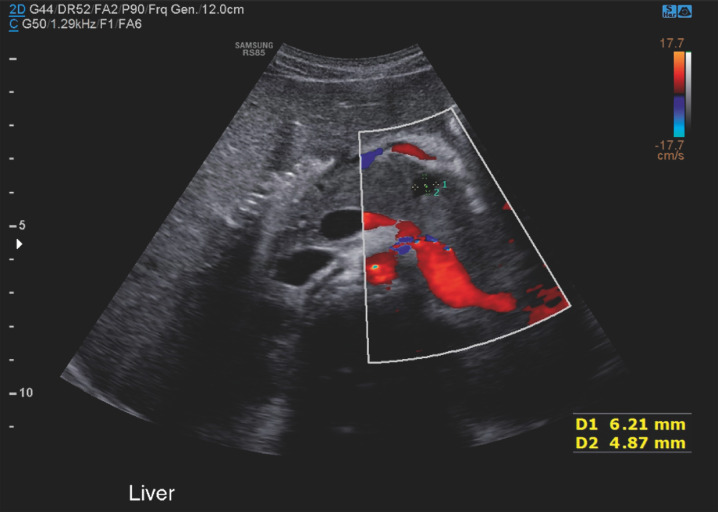
Ultrasound of the abdomen showing resolved fluid collection in the pancreas previously seen on abdominal MRI.

The use of ERCP in traumatic pancreatic injury was first reported by Hall et al. in 1986 3 . In the pediatric population there are concerns regarding ERCP including the expertise needed to cannulate small papilla and post-ERCP pancreatitis 4 . Conventionally severe pancreatic injuries involving the pancreatic duct are managed with surgery 2 . However, studies have demonstrated that ERCP can be safely performed in the pediatric population and can act either as an adjuvant or provide definitive treatment for pancreatic duct injuries in the pediatric population 5 .

Endoscopy_UCTN_Code_TTT_1AR_2AI

## Notes

Endoscopy E-Videos https://eref.thieme.de/e-videos
*E-Videos* is an open access online section of the journal *Endoscopy* , reporting on interesting cases and new techniques in gastroenterological endoscopy. All papers include a high-quality video and are published with a Creative Commons CC-BY license. Endoscopy E-Videos qualify for HINARI discounts and waivers and eligibility is automatically checked during the submission process. We grant 100% waivers to articles whose corresponding authors are based in Group A countries and 50% waivers to those who are based in Group B countries as classified by Research4Life (see: https://www.research4life.org/access/eligibility/ ). This section has its own submission website at https://mc.manuscriptcentral.com/e-videos .
